# Magnon-Assisted Magnetization Reversal of Ni_81_Fe_19_ Nanostripes on Y_3_Fe_5_O_12_ with Different Interfaces

**DOI:** 10.1021/acsnano.3c06353

**Published:** 2024-03-15

**Authors:** Andrea Mucchietto, Korbinian Baumgaertl, Dirk Grundler

**Affiliations:** # Laboratory of Nanoscale Magnetic Materials and Magnonics, Institute of Materials (IMX), École Polytechnique Fédérale de Lausanne (EPFL), 1015 Lausanne, Switzerland; ‡ Institute of Electrical and Micro Engineering (IEM), ’Ecole Polytechnique Fédérale de Lausanne (EPFL), 1015 Lausanne, Switzerland

**Keywords:** spin waves, magnons, magnetization reversal, ferrimagnet, ferromagnet, spectroscopy, magnetic interfaces

## Abstract

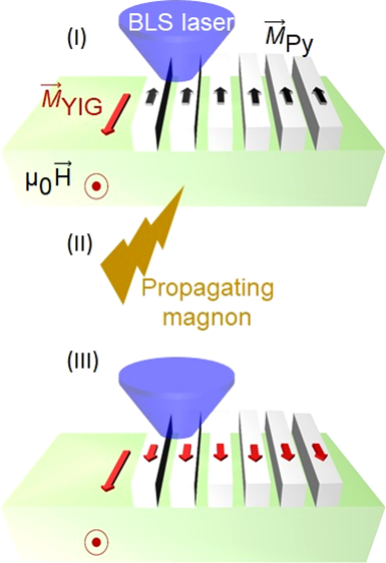

Magnetic bit writing by short-wave magnons without conversion to the electrical domain is expected to be a game-changer for in-memory computing architectures. Recently, the reversal of nanomagnets by propagating magnons was demonstrated. However, experiments have not yet explored different wavelengths and the nonlinear excitation regime of magnons required for computational tasks. We report on the magnetization reversal of individual 20 nm thick Ni_81_Fe_19_ (Py) nanostripes integrated onto 113 nm thick yttrium iron garnet (YIG). We suppress direct interlayer exchange coupling by an intermediate layer, such as Cu and SiO_2_. By exciting magnons in YIG with wavelengths λ down to 148 nm we observe the reversal of the integrated ferromagnets in a small external field of 14 mT. Magnons with a small wavelength of λ = 195 nm, i.e., twice the width of the Py nanostripes, induced the reversal at a spin-precessional power of only about 1 nW after propagating over 15 μm in YIG. Such small power value has not been reported so far. Considerations based on dynamic dipolar coupling explain the observed wavelength dependence of the magnon-induced reversal efficiency. For an increased power, the stripes reversed in an external field of only about 1 mT. Our findings are important for the practical implementation of nonvolatile storage of broadband magnon signals in YIG by means of bistable nanomagnets without the need of an appreciable global magnetic field.

Collective spin excitations in a magnetically ordered material are called spin waves (SWs) or, in quantum-mechanical terms, magnons. By means of SWs, angular momentum is transferred without electrical charge motion; hence, no Joule heating is generated. Therefore, SWs represent an alternative paradigm for signal processing at low power consumption and for a non-charge-based beyond-CMOS technology.^1−3^ In magnonic applications, microwave signals are applied to integrated coplanar waveguides (CPWs) and excite coherent SWs in the adjacent magnetic layer. Grating couplers consisting of ferromagnetic nanoelements [Figure 1(a)] have been proven to enhance the microwave-to-magnon coupling at GHz frequencies if integrated to CPWs.^4−7^ They emit and detect magnons with wavelengths λ down to below 50 nm in ferrimagnetic yttrium iron garnet (YIG).^8,9^ Wang et al. explored the spin wave emission from a ferromagnetic stripe into YIG.^10^ They explained the strong spin-wave signal in the underlying YIG by prominent dipole–dipole interaction without assuming spin currents. Recently, it has been reported that dipolar SWs reversed 100 nm-wide ferromagnetic nanostripes deposited directly on YIG after propagating over 25 μm.^11^ The magnon-induced switching of Ni_81_Fe_19_ (Py) nanostripes on YIG occurred in the linear excitation regime at a low microwave power. However, the wavelength λ used for switching remote nanostripes was a few micrometers long. Such value of λ is not adequate for nanomagnonic in-memory computing in either the linear or nonlinear excitation regime.^12−14^

**Figure 1:**
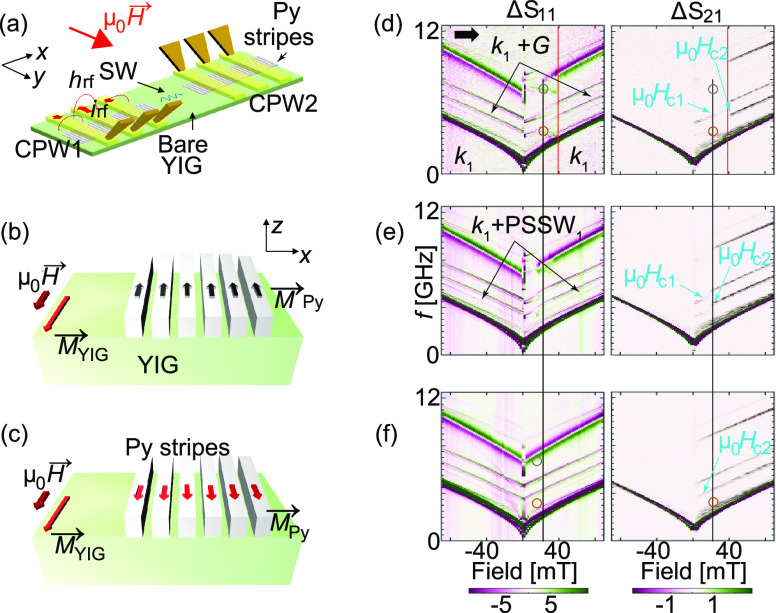
(a) Schematic device with the two CPWs, Py stripes (gratings) and microwave tips connected to a VNA. A current *i*_rf_ at frequency *f*_irr_ is injected into CPW1. The generated field *h*_rf_ excites magnons. Sketches of the (b) antiparallel (AP) and (c) parallel (P) magnetic configuration of Py nanostripes and YIG. Color-coded spectra Δ*S*_11_ (left) and Δ*S*_21_ (right) taken as a function of field from −90 mT to +90 mT on sample A for powers *P*_irr_ of (d) −30 dBm, (e) −15 dBm, and (f) 0 dBm. The horizontal arrow in (d) indicates the magnetic field sweep direction. We display Δ*S*, i.e., the difference of scattering parameters *S* that are taken at subsequent field values. Intense and dark colors indicate magnon resonances. Labels and symbols highlight specific resonances and critical fields. The black (red) vertical lines indicate 24 mT (40 mT). In (f, right) the AP branch is not resolved indicating *H*_C1_ is (close to) zero.

In this work we report remote switching of 100 nm wide Py nanostripes by magnons with λ down to 148 nm in YIG. We explore different interfaces and both the linear and nonlinear excitation regime. The Py nanostripes were integrated on an intermediate layer of either Cu or SiO_2_ on YIG. Thereby we suppressed the direct exchange coupling^15^ between Py and YIG. Using the identical nanostripe design, we compare our results to ref (11) in which an intermediate layer between Py and YIG was avoided. By using broadband spectroscopy [Figure 1(d)–(f)] and spatially resolved Brillouin light scattering (BLS) we acquire magnon spectra before and after exciting propagating magnons of different λ in YIG. We observe irreversible changes in BLS spectra which indicate reversed states of Py magnetization vectors **M**_Py_ which we attribute to magnon-induced switching in a small external field. We analyze the power absorbed by the precessing spins in YIG and find that propagating magnons whose wavelength is twice the nanostripe width show a minimum power level of about 1 nW representing the highest reversal efficiency. Our findings go beyond earlier reports in that we (i) demonstrate experimentally that dynamic dipolar coupling between Py and YIG is sufficient for magnon-induced reversal, (ii) explain the wavelength dependent reversal efficiency, and (iii) report switching by propagating magnons with a wavelength of only 148 nm. (iv) Our BLS data reveal the magnon-induced reversal in the nonlinear excitation regime which we attribute to parametrically pumped magnons. Our findings are key for the progress toward in-memory computation in linear and nonlinear nanomagnonics with materials combinations which do not require direct exchange coupling between the magnetic elements.

## Results and Discussion

We fabricated one-dimensional (1D) periodic arrays of Py nanostripes (gratings) on 113 nm thick YIG which was commercially available from the same supplier as in refs (7, 16, 17). The stripes consisted of 20 nm thick Py and were 100 nm wide. They were arranged with a period of *p* = 200 nm. The stripe lengths were consistent with ref (11) and alternated between 25 and 27 μm. The total width of the grating amounted to *w*_GC_ = 10 μm. They were fabricated on YIG with a 5 nm thick intermediate layer of either Cu (sample A) or SiO_2_ (sample B). We introduced the intermediate layers to intentionally modify the coupling between Py and YIG compared to ref (11) where Py had been deposited directly on YIG. The intermediate layers suppressed the exchange coupling. Moreover, in sample B the SiO_2_ spacer avoided the spin pumping mechanism, thus allowing for only dipolar coupling between Py and YIG. In sample A, the Cu spacer thickness was smaller than the spin diffusion length^18^ and a spin pumping related torque could occur in addition to dipolar coupling.^19^ In the following we denote the sample without an intermediate layer used in ref (11) as sample C. In the coplanar waveguides (CPWs) [Figure 1(a)], the Au lines (gaps) were 2.1 μm (1.4 μm) wide. The distance between the signal lines of two parallel CPWs was 15 μm. A finite element analysis using COMSOL Multiphysics provided the inhomogeneous radiofrequency (rf) field *h*_rf_ of the CPWs (Figure S1). Without the lattice of nanostripes, they excited and detected spin waves most efficiently with a wave vector *k* of *k*_1_ = 0.87 rad/μm. An in-plane magnetic field **H** was applied to realize specific magnetic histories and controlled different relative orientations of magnetization vectors in Py (**M**_Py_) and YIG (**M**_YIG_) [Figure 1(b),(c)]. We performed broadband measurements (Methods) of scattering parameters Δ*S*_11_ (reflection) and Δ*S*_21_ (transmission) with ports 1 and 2 of a vector network analyzer (VNA) connected to CPW1 and CPW2, respectively. We observed several resonant branches above the *k*_1_ excitation in Figure 1(d)–(f). Considering refs (5, 7, 8), the additional high-frequency branches reflected grating coupler (GC) modes such as *k*_1_ + *G*, with *G* = 2π/*p*, different orders of perpendicular standing spin waves (PSSWs), and the magnetic resonance in the Py nanostripes. The latter one was the prominent high-frequency branch in Δ*S*_11_ which started at about 10.5 GHz at −90 mT in Figure 1(d). In Figures S2 and S3 we report further spectra from which we extracted the quasi-static characteristics of samples A and B. We applied BLS microscopy (μBLS) in that we focused laser light for inelastic light scattering on Py nanostripes in the different gaps of a CPW. The laser spot diameter was about 400 nm. We note that the resonance frequency of Py nanostripes was high (low) if **M**_Py_ was parallel (antiparallel) to the applied field **H** (see below).^20^ The BLS microscopy was used to gain spatially resolved information about Py nanostripe reversal and explore the nonlinear regime which was not achieved in ref (11).

**Figure 2:**
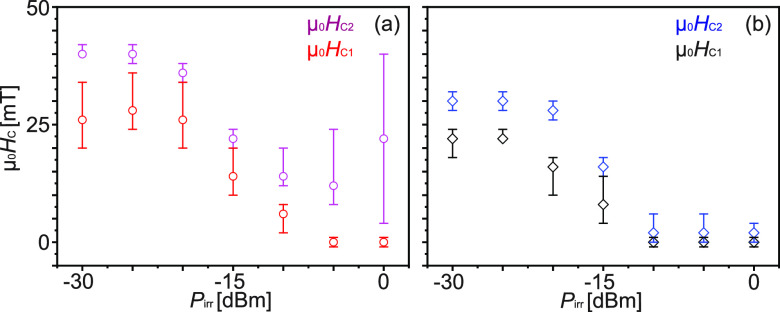
For samples (a) A and (b) B the critical fields μ_0_*H*_C1_ and μ_0_*H*_C2_ realizing 50% of the maximum signal strengths of the two relevant magnon branches are shown as a function of *P*_irr_. The error bar refers to fields needed to achieve 30% and 70% of the maximum signal strengths of branches AP and P.

For obtaining the spectra Δ*S*_11_ and Δ*S*_21_ of sample A in Figure 1(d)–(f) we applied **H** along the *y*-direction of sample A. We measured the scattering parameters in the following order: *S*_11_, *S*_21_, *S*_22_, and *S*_12_. In the following we focus on spin waves that were excited at CPW1 and propagated to CPW2; i.e., we report spectra *S*_11_ and *S*_21_, respectively. The spectra of *S*_22_ and *S*_12_ showed consistent features when considering nonreciprocity and applying an inverted magnetic history. In all of our experiments, the in-plane magnetic field **H** is applied along the longitudinal axis of the CPWs, i.e., *y*-axis in Figure 1a. The excited spin waves propagate in the YIG plane along the *x*-axis. Their wave vector is perpendicular to **H**. This configuration is known as the Damon–Eshbach (DE) configuration. We varied μ_0_*H* from −90 mT to +90 mT [indicated by the black horizontal arrow in Figure 1(d)] in steps of 2 mT. The nonreciprocal spin wave characteristics led to the large signal-to-noise ratios at positive *H*. The same measurement protocol was repeated for different VNA powers *P*_irr_ = −30, −15, and 0 dBm in Figure 1 (from top to bottom). At small power, we interpreted the branches of Figure 1(d) such that at small positive μ_0_*H* below μ_0_*H*_C1_ = 26 mT [Figure 2(a)] the magnetization vectors of YIG and Py nanostripes were antiparallel (AP) [Figure 1(b)], in agreement with Co nanostripes on YIG reported in ref (8). We defined μ_0_*H*_C1_ as the critical field at which the AP branch possessed 50% of its maximum intensity (indicated by the arrows in Figure 1(d),(e). In this low-field regime, the branch with negative slope d*f*/d*H* in Δ*S*_11_ [marked by a gray circle in Figure 1(d)] was attributed to the ferromagnetic resonance inside the Py nanostripes. Their magnetization vectors **M**_Py_ pointed still in the −*y*-direction and against the applied positive field **H**. They were antiparallel also with **M**_YIG_ as YIG had a coercive field ≤2 mT. At small applied power *P*_irr_ = −30 dBm, several of the grating coupler (GC) modes gained abruptly a pronounced signal strength at 40 mT (indicated by the red dashed line). We attributed this observation to the critical field μ_0_*H*_C2_ [Figure 2(a)] at which the reversal of the Py nanostripes underneath CPW2 (i.e., the detector CPW) occurred. We defined μ_0_*H*_C2_ as the critical field at which the P branch possessed 50% of its maximum intensity (indicated by arrows in Figure 1d to f). For μ_0_*H* > 40 mT, all of the detected branches in Figure 1(d) were similar to the ones at the correspondingly large negative fields. These branches indicated that the magnetization vectors of Py nanostripe lattices underneath both CPWs were now parallel (P) to **H** and **M**_YIG_. Correspondingly, the transmission data showed the richest spectra of grating coupler modes [Figure 1(d) on the right].

For the spectra Δ*S*_11_ shown in Figure 1(e), we used a larger power *P*_irr_ of −15 dBm. In the transmission spectra Δ*S*_21_ [right panel in Figure 1(e)], the AP branch ended at a smaller field value μ_0_*H*_C1_ = 14 mT and the region P started near μ_0_*H*_C2_ = 22 mT instead of 40 mT. The Py nanostripes underneath CPW1 and CPW2, respectively, experienced a smaller field region of antiparallel alignment with **M**_YIG_ compared to Figure 1(d). This observation indicated that the larger VNA power *P*_irr_ used for broadband spectroscopy led to the reversal of Py nanostripes underneath both CPW1 and CPW2.

The onset of the P region occurred at an even smaller *H* in Figure 1(f) when a *P*_irr_ of 0 dBm was used. The branches attributed to grating coupler modes in the P region showed a weak signal strength already at μ_0_*H* = 2 mT which increased with increasing *H*. This means that close to zero field the magnetization vector **M**_Py_ underneath CPW1 pointed into the +*y*-direction (P configuration), i.e., μ_0_*H*_C1_ ≤ 2 mT. The reversal field of the Py nanostripes under CPW1 of sample A was hence reduced by about 26 mT when applying *P*_irr_ = 0 dBm (1 mW) compared to *P*_irr_ = −30 dBm (1 μW). Such a large reduction of μ_0_*H*_C1_ was not reported in ref (11) (sample C) which had Py directly deposited on YIG.

To characterize the power-dependent switching field distribution for samples A and B (Figure 2) we adopted the methodology developed in ref (11). We evaluated VNA spectra taken at many different powers *P*_irr_ and analyzed the field-dependent signal strengths of the first grating coupler branch in the AP and P state. In such experiments, we applied *i*_rf_ covering a broad frequency regime from about 10 MHz to 20 GHz. Assuming that the magnon mode for most efficient switching resided in this frequency regime, we obtained the minimum critical field values for reversal at a given *P*_irr_. At each value of *P*_irr_, we extracted the critical field values μ_0_*H*_C1_ and μ_0_*H*_C2_ that corresponded to 50% of the maximum signal strengths of the AP and P branches, respectively (symbols in Figure 2). The difference (*H*_C2_ – *H*_C1_) reflected the distribution of switching fields of nominally identical Py nanostripes underneath the two CPWs. In sample A (Figure 2a), the switching fields were distributed over a larger field range than in sample B (Figure 2b) for *P*_irr_ < −20 dBm.

We first consider the critical fields μ_0_*H*_C1_ extracted from the AP branches of both samples A and B. At low power, *P*_irr_ ≤ −25 dBm, μ_0_*H*_C1_ is comparable within error bars in both samples. μ_0_*H*_C1_ decreases to 0(±1) mT in sample A (B) when *P*_irr_ ≥ −5 dBm (−10 dBm). We now focus on the critical fields of the P branch, *i.e. μ*_0_*H*_C2_. For *P*_irr_ < −20 dBm the critical field μ_0_*H*_C2_ is larger for sample A than for sample B (cf. Figure 2a and 2b). μ_0_*H*_C2_ decreases as *P*_irr_ increases to −5 dBm. μ_0_*H*_C2_ is reduced to 2 mT in sample B when *P*_irr_ ≥ −10 dBm and it maintains this small value at larger *P*_irr_. This is the smallest value so far detected for the reversal of Py nanostripes on YIG induced by propagating magnons. This finding is one of the key achievements of this work. Near-zero critical fields were not be observed in ref (11).

For comparison, in sample A, the critical field μ_0_*H*_C2_ is −15 mT at −5 dBm. At the largest *P*_irr_, it has increased again (Figure 2a) and reaches ∼22 mT. Considering ref (11), we attribute this increase in critical fields of Py nanostripes underneath CPW2 at high *P*_irr_ to the nonlinear regime of magnon excitation underneath CPW1 with enhanced magnon scattering. Because of the scattering processes and increased losses^21^ the magnon amplitudes after 15 μm are below the threshold for complete reversal of the nanostripe array under CPW2. The incomplete reversal at high power is observed for samples A and C where spin pumping is allowed. In sample B (Figure 2b) we do not observe an increase in critical fields at large powers. Instead, we find the largest reduction in critical fields *H*_C1_ and *H*_C2_ in sample B. Here, a 5 nm thick SiO_2_ spacer rules out that spin pumping is relevant for the efficient magnon-induced reversal. We note that the insertion of both the SiO_2_ and Cu spacer excludes the transfer of exchange magnons which was assumed in refs (22−25). Our experiments highlight the importance of dynamic dipolar coupling between Py and YIG when developing a microscopic understanding of the magnon-induced reversal mechanism.

**Figure 3:**
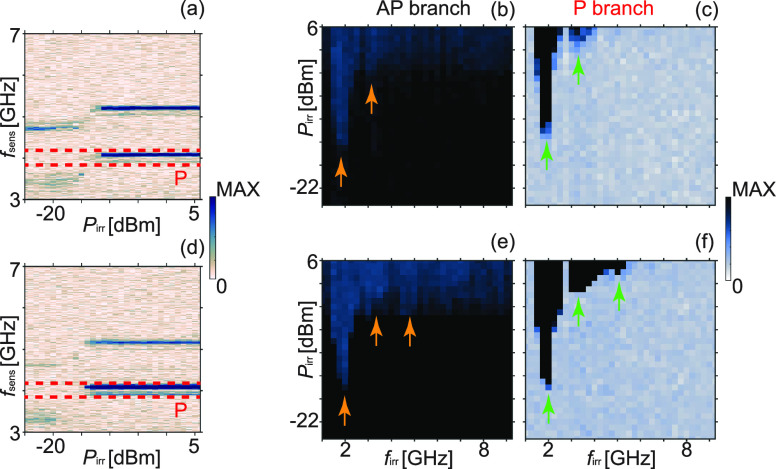
(a) Mag(*S*_21_) recorded on sample A (Cu spacer) between *f*_sens_ = 3 and 7 GHz at +14 mT with a power of −25 dBm after applying a microwave signal with *f*_irr_ = 1.75 GHz to CPW1 for increasing power *P*_irr_. Switching yield maps at +14 mT for sample A displaying color-coded (b) Mag(*S*_11_) and (c) Mag(*S*_21_) integrated as a function of *f*_sens_ for the AP and P branch, respectively. The frequency integration range for the P branch is highlighted by the red dashed lines in (a). To extract the switching yield map for the AP branch, the first GC mode branch in the Mag(*S*_11_) spectrum is used. Panels (d) to (f) show the corresponding data set for sample B (SiO_2_ spacer). Arrows indicate local minima in the power threshold inducing stripe reversal by specific magnons discussed in the text.

**Figure 4:**
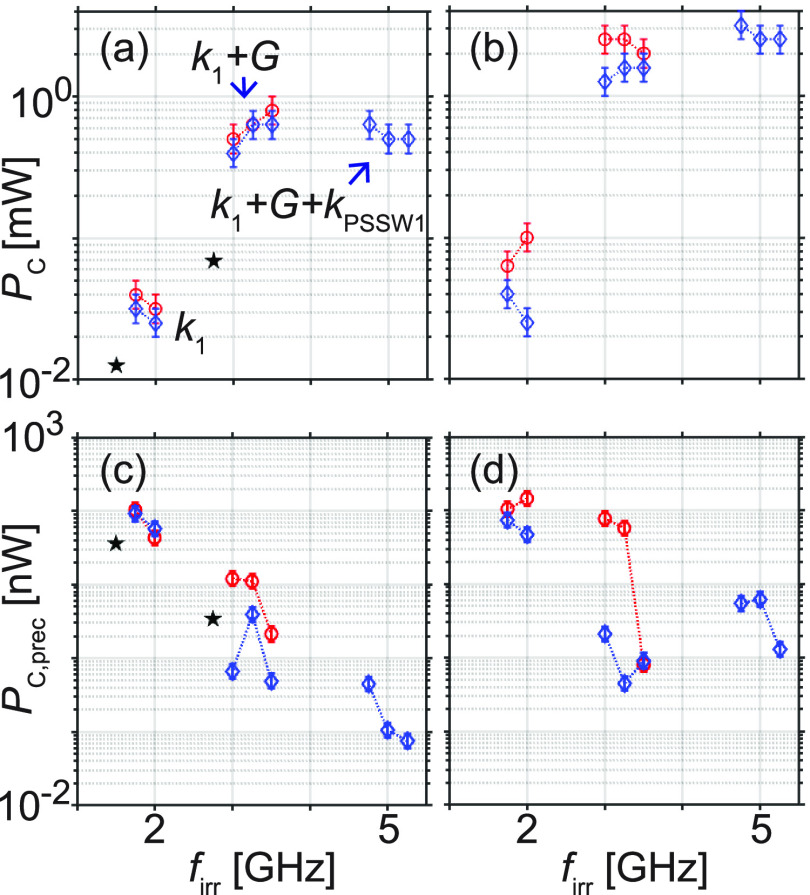
For samples A (red), B (blue), and C (black) the critical powers (a) *P*_C1_ and (b) *P*_C2_, (c) *P*_C1,prec_ and (d) *P*_C2,prec_ are depicted for irradiation frequencies *f*_irr_ in half-logarithmic graphs. In (a) we label magnon modes by *k*_1_, *k*_1_ + *G* and *k*_1_ + *G* + *k*_PSSW1_. Connecting lines are guide to the eyes.

To quantify the power level at which a specific spin wave mode in YIG reversed nanostripes we followed the concept of switching yield maps (Methods) introduced in ref (11) (Figure 3). The samples were first saturated at −90 mT applied along the *y*-axis. Then, the field was gradually increased to +14 mT and kept constant. We provided powers *P*_irr_ ranging from −25 to +6 dBm with +1 dBm steps within a 0.25 GHz wide frequency window starting at a specific frequency *f*_irr_. After each power step and corresponding irradiation for 1 ms, the VNA power level was reduced to −25 dBm and the transmitted signal (*S*_21_) was recorded as a function of frequency *f*_sens_ ranging from 3 to 7 GHz. Figure 3 displays such data sets in panels (a) and (d) as well as grayscaled switching yield maps performed at +14 mT for sample A (top row) and sample B (bottom row). The maps labeled by AP (P) branch in Figure 3(b) and (e) (Figure 3(c) and (f)) reflect the reversal of Py nanostripes underneath CPW1 (CPW2) of samples A and B, respectively. From these maps, we extracted the critical power levels for magnon-assisted switching at CPW1 and CPW2 that we denote by *P*_C1_ and *P*_C2_, respectively. In Figure 4, we particularly display the critical power values extracted near the local minima indicated by arrows in Figure 3 reflecting modes *k*_1_, *k*_1_ + *G* and *k*_1_ + *G* + *k*_PSSW1_ (from left to right).

When exciting the *k*_1_ mode near 2 GHz in sample A and B at +14 mT, we require *P*_C1_ between 30 and 40 μW for the reversal of 50% of the nanostripes below CPW1 [red symbols in Figure 4(a)]. This power value is only about a factor of 3 larger than the one of sample C published in ref (11) [black symbol near 1.5 GHz in Figure 4(a)]. For all samples, *P*_C1_ and *P*_C2_ increase with increasing mode frequency. For the reversal underneath CPW1 by means of the GC mode *k*_1_ + *G* (excited between 2.75 and 3.5 GHz) VNA powers *P*_C1_ of 400 to 800 μW are required. The reversal of Py nanostripes underneath CPW2 is achieved at a further increased power level *P*_C2_ of up to 2.5 mW. For sample C, the *P*_C2_ was larger than 3 mW and not determined.

To compare different samples, it is instructive to consider the power values *P*_C1, prec_ [Figure 4(c)] and *P*_C2, prec_ [Figure 4(d)] which quantify the power absorbed by the spin-precessional (prec) motion in YIG at the emitter CPW (Experimental Methods). These values consider that only part of the rf power applied by the VNA is absorbed by the spin system and converted into magnons. These values, taken in the same field, allow us to compare the different samples independent of the individual efficiency of microwave-to-magnon transduction. In case of the long-wavelength modes *k*_1_ existing near 2 GHz in samples A and B, we observe reversal at power levels *P*_C1, prec_ between 4 to 10 nW [Figure 4(c)]. *P*_C2, prec_ for modes *k*_1_ is only slightly larger attributed to a weak decay of the magnon mode between emitter and detector CPW. At larger excitation frequencies *f*_irr_ between 3 and 3.5 GHz corresponding to the first GC mode resonance *k*_1_ + *G*, power values *P*_C1,prec_ are smaller by up to 2 orders of magnitude compared to modes *k*_1_ in Figure 4(c). Here, sample B realizes the smallest values *P*_C1,prec_ down to about 0.5 nW. Note that despite larger coercivities in sample A the magnon-induced reversal underneath CPW1 via mode *k*_1_ + *G* is realized at a lower power than in sample C with the direct interface between Py and YIG. A similar small value of about 1 nW is found in Figure 4(d) for reversal underneath CPW2, suggesting a weak decay of magnon amplitudes after a path of 15 μm. The key finding of Figure 4(d) is that mode *k*_1_ + *G* in sample B is the most efficient in terms of *P*_C2,prec_ and nanostripe reversal underneath CPW2. Considering its intermediate layer to be an insulator (SiO_2_) the dipolar coupling between magnons in YIG and Py provides the torque for the reversal.

We note that in sample B we observe nanostripe reversal by a further mode with wavelength λ = 148 nm corresponding to a wavevector  (*k*_PSSW1_ is the first quantized magnon mode across the YIG thickness). When exciting this mode in the frequency range 4.75–5.25 GHz, we extract *P*_C2,prec_ = 1.3–5.4 nW. These power values are increased compared to *P*_C2,prec_ found near 3 GHz. For sample B, we observe the smallest spin-precessional power *P*_C1,prec_ for reversal in Figure 4(c) at 5.25 GHz. We explain the small value by the combined effect of magnon-induced reversal and microwave-assisted switching near the eigenresonance of the Py nanostripes underneath CPW1 before their reversal at +14 mT. For the nanostripes underneath CPW2 (*P*_C2,prec_) the microwave-assisted switching does not play a role due to their large separation from the CPW1 attached to the rf source. We have repeated the same experiments at +20 mT as well. The power levels *P*_C_ and *P*_C,prec_ for magnon-induced reversal are reported in the Supporting Information and show consistent characteristics.

**Figure 5:**
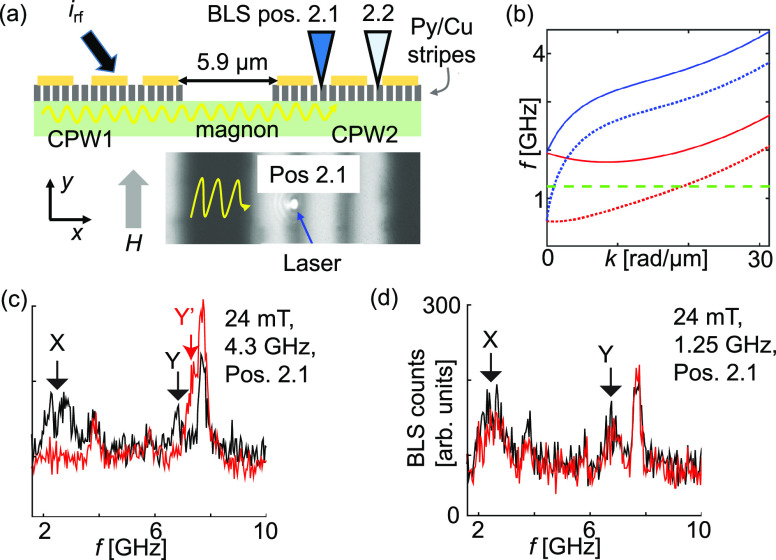
(a) Sketched cross-section of the device (top). The CPW lines (yellow) are on top of the stripes (dark gray) which have been fabricated on YIG (green). In BLS we detected thermally excited magnons in Pos. 2.1 (microscopy image) and 2.2. (b) Magnon dispersions in the thin YIG at 24 (solid lines) and 2 (dotted lines) mT calculated via the Kalinikos–Slavin formalism for two limiting configurations, i.e., Damon–Eshbach (blue lines) and backward volume (red lines) configuration. The horizontal dashed green line indicates 1.25 GHz which is below (inside) the magnon band at 24 mT (2 mT). Magnon spectra in Pos 2.1 at 24 mT before (black) and after (red) applying *i*_rf_ to CPW1 for *P*_irr_ = 16 dBm with (c) 4.3 GHz and (d) 1.25 GHz. Labels X, Y, and Y′ indicate characteristic resonant modes.

In the following, we apply microfocus BLS to sample A [Figure 5(a)] and gain spatially resolved information about the magnon modes that modify the magnetization vectors **M**_Py_ of Py stripes. We do not evaluate absolute power values here as the BLS setup has not allowed for calibration and CPWs were wire-bonded.

We discuss BLS spectra reflecting the incoherent magnons excited at room temperature. We compare spectra taken before (black curves) and after (red curves) applying microwaves to CPW1. We explore different fields *H* modifying the spin-wave dispersion relation in YIG [Figure 5(b)] and different *f*_irr_. The laser wavelength (power) was 473 nm (0.8 mW). Given the laser spot diameter of about 400 nm, we collected the Stokes’s signal of magnons from up to two Py nanostripes and the underlying YIG. Each spectrum in Figure 5(c),(d) had an acquisition time of approximately 2 h.

The spectrum shown as the black curve in Figure 5(c) displays magnon resonances existing in the gap of CPW2 in Pos. 2.1 for μ_0_*H* = 24 mT after saturation along the – *y*-direction using μ_0_*H* = −84 mT and before applying *i*_rf_ to CPW1. The frequencies of resonances marked X and Y indicate that the Py nanostripes are antiparallel to **H** [Figure 1(b)]. They are consistent with the frequencies marked by brown and gray circles, respectively, in Figure 1(d). After applying *i*_rf_ at *f*_irr_ = 4.3 GHz to CPW1 with a nominal irradiation power *P*_irr_ of up to 39.8 mW (16 dBm) the red spectrum was obtained at the same position. Due to wire-bonded connections, we expected the power in CPW1 to be a few dB lower than the nominal value. The red spectrum is markedly modified compared to the black curve: the resonance peaks X and Y reduced to the noise level, and a higher frequency resonance Y′ was resolved. The peak Y′ in the red spectrum was consistent with the branch existing in the P configuration of the sample above the gray circle in Figure 1(f). The microwave current applied to CPW1 with *f*_irr_ = 4.3 GHz hence led to the reversal of Py nanostripes at the remotely located CPW2.

**Figure 6:**
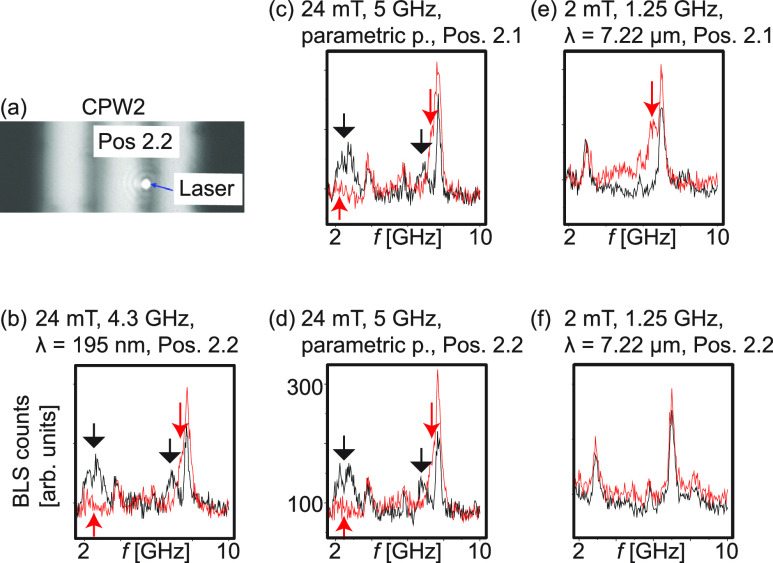
(a) Optical image when positioning the laser at Pos. 2.2. (b) Magnon spectra taken in Pos. 2.2 at 24 mT before (black) and after (red) applying *i*_rf_ to CPW1 with *f*_irr_ = 4.3 GHz. The reversal of Py nanostripes by magnons *k*_1_ + *G* is evidenced. Magnon spectra in (c) Pos. 2.1 and (d) Pos. 2.2 before (black) and after (red) applying *i*_rf_ with *f*_irr_ = 5 GHz at CPW1. The black (red) arrows highlight characteristic modes (changes). Thermal magnon spectra in (e) Pos. 2.1 and (f) Pos. 2.2 before (black) and after (red) emitting magnons with *k*_1_ by applying *i*_rf_ with *f*_irr_ = 1.25 GHz at CPW1. In all these experiments the irradiation power was *P*_irr_ = 16 dBm. The legends list relevant parameters and highlight the parametric pumping (p.) experiments.

To investigate if heating of CPW1 by *i*_rf_ initiated the reversal we followed the same measurement protocol as applied in Figure 5(c) but changed the rf signal frequency to 1.25 GHz. The signal *i*_rf_ was applied for 2 h, before taking the red spectrum in Figure 5(d). The red spectrum is found to contain the identical resonances as the black spectrum; i.e., **M**_Py_ was not changed by applying an rf signal at 1.25 GHz. We explain the different spectra (red) in panels (c) and (d) of Figure 5 by the dispersion relation of YIG at 24 mT displayed in Figure 5(b). At *f*_irr_ = 1.25 GHz (green dashed line), magnons are not emitted into YIG as all allowed magnon bands reside at higher frequencies. This is different for an *f*_irr_ of 4.3 GHz. Here, a Damon–Eshbach (DE) mode is allowed and excited at CPW1. It propagates to CPW2 as evidenced by the transmission spectra shown in Figure 1. The allowed mode is grating coupler mode *k*_1_ + *G* with λ = 195 nm. The characteristic resonance Y’ in the red spectrum of Figure 5(c) evidences the reversal of Py nanostripes in Pos. 2.1 by the propagating magnon mode. In Pos. 2.2 located a few micrometers farther away from CPW1 (Figure 6a), we detected a modified spectrum (red) at 24 mT as well (Figure 6(b)). Hence, the reversal of Py nanostripes was induced in both gaps by the short-wave magnon mode *k*_1_ + *G* excited at CPW1 at *f*_irr_ = 4.3 GHz.

When applying a microwave signal with *f*_irr_ = 5 GHz to CPW1 (after again initializing sample A at −84 mT), we observed modified spectra (red) taken at Pos. 2.1 [Figure 6(c)] and Pos. 2.2 [Figure 6(d)]. Note that the directly excited grating coupler mode did not exist at 5 GHz. Still, the finding is different from the experiment conducted with *f*_irr_ = 1.25 GHz in Figure 5(d). We attribute the observed reversal of Py nanostripes to magnons, which were excited by parametric pumping at CPW1 (Supporting Information). Their frequency reads *f*_m_ = *f*_irr_/2 = 2.5 GHz, which was above the *k*_1_ resonance (*f*_*k*_1__ = 2.3 GHz) at 24 mT and inside the allowed magnon band for propagation. Such magnons, hence, reached CPW2 and could explain the observed reversal. We note that the phase-sensitive voltage detection of the VNA experiment does not allow us to show the magnons created by parametric pumping because of their shifted frequency. We resolve them by BLS as presented in detail in Figure S7.

We performed experiments also at 2 mT after initializing the sample at −84 mT. In Pos. 2.1 [Figure 6(e)] we observed that the magnon spectrum (red) was modified after applying *i*_rf_ with *f*_irr_ = 1.25 GHz. At 2 mT, spin waves at this small frequency were allowed [dotted lines in Figure 5(b)] and possessed a wave vector *k*_1_ with λ = 7222 nm. Excited at CPW1, the magnon mode changed the Py nanostripes underneath CPW2 at Pos. 2.1, but not at Pos. 2.2 [Figure 6(f)]. We assume that at the small field the excitation of the *k*_1_ mode was in the nonlinear regime as well but additional parametric pumping did not take place at the small frequency. Instead, the amplitude of magnons decayed due to enhanced scattering and was below the threshold for reversal in Pos. 2.2. The excitation of propagating magnons with a too high microwave power hence led to an incomplete reversal of nanostripes below CPW2. This finding is consistent with the nonmonotonous variation of critical fields with applied microwave power reported in ref (11) and as shown in Figure 2. BLS studies on magnon-induced reversal in sample B are shown in Figure S8 and support the findings reported for sample A.

Our assumption that dipolar coupling is important for reversal is supported by the theoretical work of Yu et al.^26^ They considered an array of ferromagnetic nanowires on top of a YIG film with an intermediate insulating Al_2_O_3_ layer. Such a hybrid system is similar to our sample B which contains an SiO_2_ layer between Py and YIG. In ref (26) the authors concluded that spin currents (magnons) in YIG could be diverted into ferromagnetic nanowires which were magnetized antiparallel to YIG. They evaluated only the dipolar coupling between magnons in YIG and the ferromagnet. The authors speculated that these spin currents which are solely due to dipolar coupling explain the magnetization reversal by magnons,^26^ i.e., the phenomenon which we report in the present manuscript.

In the following, we discuss the possible origin for the observed variation of spin-precessional power for magnon-induced reversal. We focus on Figure 4(d), where we exclude direct microwave-assisted switching of nanostripes by the applied rf signal. In sample B, we observe the smallest spin-precessional power when the propagating magnons exhibit a wavelength of 195 nm underneath the gratings.^7^ This value is (very close to) twice the width of a Py nanostripe. We argue that such a relation between the magnon wavelength and nanostripe width ensures the highest possible repetition rate by which a maximum in the dipolar stray field of a DE mode exerts a torque on the Py magnetization vector **M**_Py_. For a shorter magnon wavelength a partial cancellation of the dynamic dipolar field occurs underneath a nanostripe, and the dynamic dipolar coupling is reduced. For a long wavelength the repetition rate is small by which the maxima of the dynamic stray field pass by the nanostripe and produce the relevant torque. These considerations motivate the observed minimum in Figure 4(d) as a function of *f*_irr_ modifying the magnon wavelength. In ref (15), the authors investigated the coupling of ferromagnet/YIG hybrid structures with intermediate layers consisting of either a 1.5 nm thick insulating AlO_*x*_ layer or a 5 nm thick Cu layer. Only the latter spacer allowed for dynamic exchange coupling. The slightly increased power levels needed for reversal in sample A incorporating the Cu spacer might indicate that the additional dynamic exchange coupling reduced the total torque for the reversal. Further studies on stripes with different spacer layers and of e.g. different lengths and widths are needed to engineer their own eigenresonance frequency and explore in detail the hypothesis of a wavelength-dependent reversal mechanism drawn from the presented experiments.

## Conclusions

We reported a magnon-induced reversal in Py/YIG hybrid structures with different intermediate layers. We quantified and compared the power values for magnon-induced switching. Reversal of 100 nm wide Py stripes was achieved by means of propagating magnons with wavelengths ranging from 148 to 7222 nm. Their excitation was realized in both the linear and nonlinear regime. The nonlinear parametric pumping was evidenced by local BLS microscopy. In an external field of 14 mT a spin-precessional power of the order of 1 nW was enough to reverse the up to 27 μm long Py nanostripes after magnon propagation over 15 μm. The absence of interlayer exchange coupling due to a spacer layer between Py and YIG led to nanostripes with partly enhanced coercive fields compared to Py stripes directly integrated on YIG. The enhanced coercive fields are advantageous in terms of nonvolatile memory of magnon signals. Importantly, with increasing power, we achieved a reduction of switching fields of nanostripes to (nearly) zero mT. Our results promise that nonvolatile magnon-signal storage in magnetic bits is feasible for wavelogic circuits and neural networks performing computational tasks at different magnon frequencies. Considerations based on dynamic dipolar coupling suggest that the power for magnon-induced storage might be minimized when the width of the magnetic bit equals half of the wavelength of the magnon.

## Experimental Methods

### A Sample Fabrication

Devices are fabricated on 113 nm thick YIG originating from the same wafer. The YIG had been deposited by liquid phase epitaxy on a 3 in. wafer and purchased from the company Matesy GmbH in Jena, Germany. The spacer was fabricated by DC sputtering of 5 nm thick Cu on YIG. Then 20 nm thick Py (Ni_81_Fe_19_) was deposited via electron beam evaporation on the YIG. The gratings were written with electron beam lithography (EBL) using hydrogen silsesquioxane as negative resist and then transferred into the Py/Cu by ion beam etching. We etched both layers of Py and Cu. CPWs were fabricated via lift-off processing after EBL and Ti/Au (5 nm/120 nm) evaporation. For sample B, the SiO_2_ layer was deposited by e-beam evaporation, and the following steps to fabricate stripes and CPW were unchanged.

### B Broadband VNA Spectra

The broadband spectroscopy data Δ*S*_*αβ*_ (α, β = 1, 2) are obtained by nearest-neighbor subtraction of raw linear magnitude signals, i.e., Δ*S*_*αβ*_(*f*, *H*_i_) = *S*_*αβ*_(*f*, *H*_i+1_) – *S*_*αβ*_(*f*, *H*_i_). The magnetic field step is 2 mT. The linear magnitude signal Mag(*S*_*αβ*_) is obtained from the quadrature sum of the real (Re(*S*)) and imaginary (Im(*S*)) parts of median-subtracted signals. Re(*S*) (Im(*S*)) is obtained by the raw real (imaginary) part after removing, at each measured frequency, its median value across all applied magnetic fields.

### C Switching Yield Maps

To build switching yield maps (Figure 3) we followed the methodology described in ref (11) to get μ_0_*H*_C_ = 14 mT by the emission of the magnon mode *k*_1_ (in ref (11) this field *H*_C_ was labeled *H*_C2_). We required (−12 ± 1) dBm (63.1 μW) at CPW1. We compare this value to 58.4 μW needed in ref (11). The separation between CPW1 and CPW2 was larger by 20 μm in ref (11) compared to the present samples. However, the previously reported critical field was only +28 mT compared to +40 mT in Figure 1(d). This larger coercive field of nanostripes with the Cu underlayer used here might explain that a similar power level for reversal was needed though the propagation length of magnons was shorter. To characterize the critical power levels featuring the switching at CPW1 (CPW2) we focus on the frequency branch 4.5–4.7 (3.9–4.1) GHz of the *S*_11_ (*S*_21_) spectra (cf. Figure 3).

To evaluate the critical precessional power *P*_C,prec_ we first extract the minimum critical power *P*_C_ for the same frequency. The overall irradiation frequency *f*_irr_ range is divided into subintervals of 250 MHz width. We record *P*_C_ and the frequency subinterval *δf*_P_C__ that achieves *P*_C_. These measurements are conducted with a 1 kHz bandwidth and 250 MHz frequency resolution. Examples of such measurements are reported in Figure 3. The magnetic field is 14 mT. To obtain the relevant Mag(S_11_) signal, we acquire field-dependent reflection spectra with 0.1 kHz bandwidth and 3.3 MHz frequency resolution. The VNA power for these measurements is labeled *P*_b_. *P*_b_ equals −25 (−10) dBm for data sets that are analyzed to evaluate *P*_C1,prec_ (*P*_C2,prec_). The magnetic field is swept from −90 mT to positive fields larger than μ_0_*H*_C2_. With these data sets, we define for both real and imaginary parts a median value across all applied magnetic fields at each frequency point. Then the linear magnitude signal Mag(*S*_11_) is constructed as described in paragraph B of the Methods section. We focus on the frequency range defined by previously found *δf*_P_C__ and consider the reflection spectrum in the same range. Inside this frequency range we identify the frequency value f_*_ that achieves the local maximum of Mag(*S*_11_): . This represents the maximum absorbed energy by the spin system. The critical spin precessional power is then evaluated by *P*_C,prec_ = .

### D BLS Measurement Protocol

To acquire the BLS spectra, we initialize the system by applying −84 mT with a permanent magnet and then gradually increase the field to reach the targeted positive value. In so doing, the system reaches the AP state. Thermal magnon spectra are acquired before injection of any rf signal at CPW1. We apply the rf signal at CPW1 at a fixed frequency for increasing nominal powers. At each power step, we recorded the BLS signal while having the rf on. The rf irradiation at each power level is approximately 2 h long. At the end of the experiment, after switching off the rf generator, the thermal magnon spectra is measured again and compared to the one acquired in the ’as-prepared’ AP state. To minimize spatial drift and maintain the same position of the laser spot, we use a feedback system with image recognition acting every 5 min. For the BLS experiments, the sample is wire-bonded to a printed circuit board. The power levels that we discussed for BLS measurements are meant as nominal values.

## Data Availability

The data sets generated and/or analyzed during the current study are available from the corresponding author on reasonable request.

## Supporting Information Available

Numerical evaluation of the excitation spectrum of the CPW inhomogeneous dynamic field conducted by combining COMSOL simulation and FFT analysis. Broadband reflection spin wave spectra at *P*_VNA_ = −25 dBm. Protocol for evaluation of nearly zero critical fields for nanomagnet reversal. Experimental data sets acquired with Brillouin light scattering microscopy (μBLS) at the emitter CPW (CPW1) investigating magnon-induced magnetization reversal of the nanomagnets beneath CPW1. Critical power levels at +20 mT of samples A and B. μBLS data sets of the Py nanostripes during continuous-wave excitation, at different power levels, of multiple magnon modes in the underlying YIG. μBLS experiments reporting magnon-induced magnetization reversal for another device with hybrid interface Py/SiO_2_/YIG. Inductive broadband spectroscopy measurements at −25 dBm acquired for samples A, C, and B for comparison of reflection and transmission spectra (PDF)

## Notes

Mucchietto, A.; Baumgaertl, K.; Grundler, D. Magnon-assisted Magnetization Reversal of Ni_81_Fe_19_ Nanostripes on Y_3_Fe_5_O_12_ with Different Interfaces. *arXiv***2023**, 2312.15107. 10.48550/arXiv.2312.15107 (accessed February 28, 2024).
