# Supplementary material for: Magnon-Assisted Magnetization Reversal of Ni81Fe19 Nanostripes on Y3Fe5O12 with Different Interfaces
Source: ACS Nano. 2024 Mar 15;18(12):8641–8. doi: 10.1021/acsnano.3c06353 (PMC10976964; doi:10.1021/acsnano.3c06353)
Supplement: Supplementary file 1 — nn3c06353_si_001.pdf [file nn3c06353_si_001.pdf]

Magnon-assisted magnetization reversal of $Ni_{81}Fe_{19}$ nanostripes on $Y_3Fe_5O_{12}$ with different interfaces

Andrea Mucchietto,† Korbinian Baumgaertl,† and Dirk Grundler†,‡

†Laboratory of Nanoscale Magnetic Materials and Magnonics, Institute of Materials (IMX), Ecole Polytechnique Fédérale de Lausanne (EPFL), Lausanne, Switzerland

‡Institute of Electrical and Micro Engineering (IEM), EPFL, Lausanne, Switzerland

SUPPORTING INFORMATION

29 February 2024

1. Inhomogeneous dynamic field of the CPW

Finite element analysis based on COMSOL Multiphysics is performed to obtain the wavevector-dependent spectrum of the inhomogeneous dynamic field generated by the CPW. This allows to understand which wavevectors are most efficiently excited by the CPW. The maximum of the spectrum is realized at $k = k_1 = 0.87$ rad/µm.

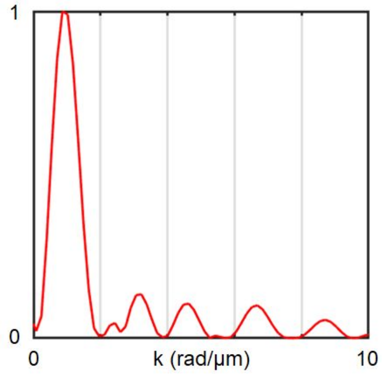

Fig. S1: Fast Fourier Transform (FFT) spectrum of the inhomogeneous dynamic field of the CPW.

2. Magnetic properties of samples A, B, and C extracted from reflection spectra

We have analyzed VNA reflection spectra Mag(S_{11}) taken at P_{VNA} = -25 dBm (Fig. S2) to extract coercive field values of Py nanostripes. We chose this power level for comparison with the measurements in Ref. [1]. We studied their resonance branches and integrated their intensities within a 400-MHz-broad range for different fields and obtained the integrated intensity as a function of the applied field. For sample A, we analyzed the branches AP and P (see labels) that were originally studied in the published work [1]. From them, we extracted the critical field values $\mu_0 H_{C1,1}$ and $\mu_0 H_{C1,2}$, respectively, that corresponded to the critical fields at which the two specific branches possessed 50% of the integrated intensity. These values reflected the switching field distribution of nanostripes below CPW1. We also extracted the critical fields $\mu_0 H_{30\%,1/2}$, and $\mu_0 H_{70\%,1/2}$ that realized 30%, and 70% of the integrated intensity, respectively. These values reflect the error bars in Fig. S3.

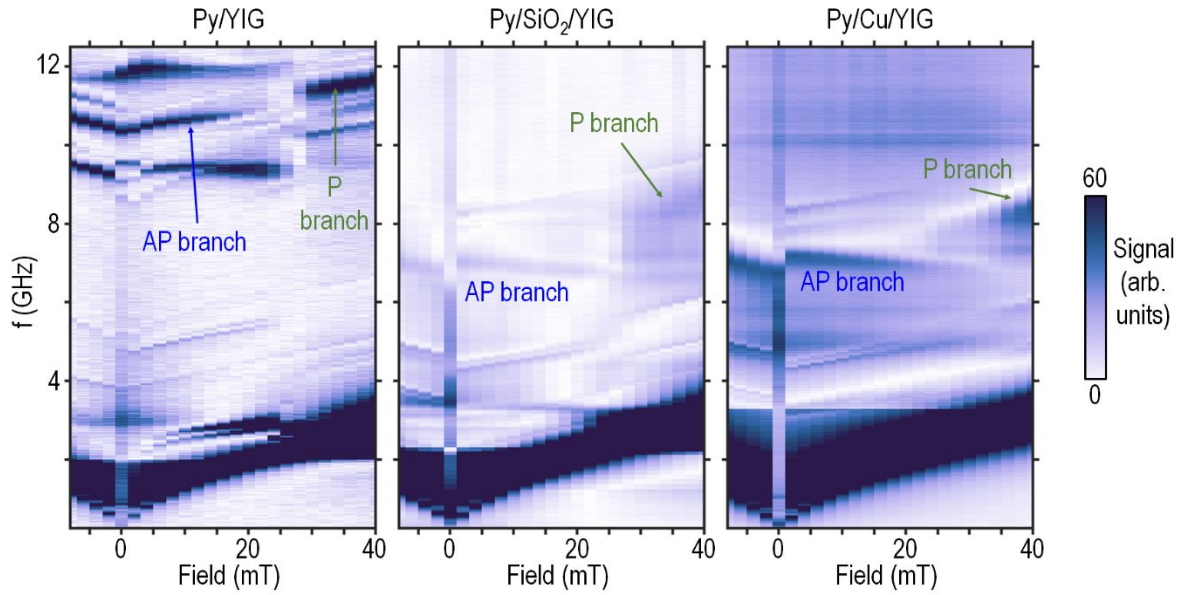

Figure S2: Reflection spectra Mag(S_{11}) acquired at P_{VNA} = -25 dBm. The AP (P) branch that we analyzed to extract the critical fields is indicated in blue (green). We show samples C, B, A from left to right.

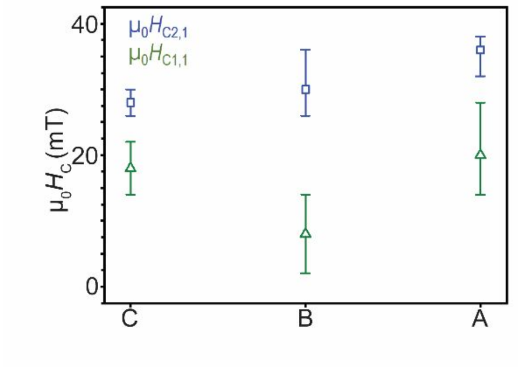

Fig. S3: Fields $\mu_0 H_{C1,1}$ ($\mu_0 H_{C2,1}$) in green (blue) for samples C, B and A characterizing the distribution of coercive fields of Py nanostripes beneath the emitter CPW1. Measurements were conducted at P_{VNA} = -25 dBm.

The mean coercive field of nanostripe arrays under CPW1 is given by $\mu_0 \langle H_{C1} \rangle = \frac{\mu_0 H_{C1,2} + \mu_0 H_{C1,1}}{2}$. For sample B, the mean coercive field is found to be the smallest one of the three samples.

3. Switching field reduction to 0 mT in transmission spectra

We focus on AP and P branches in Mag(S_{21}) transmission spectra to extract $\mu_0 H_{C1}$ and $\mu_0 H_{C2}$, respectively that are both reported in Fig. 2 of the main text. The following protocol is applied to each branch. For a fixed VNA power P_{irr}, the signal intensity Mag(S_{21}) is integrated over the frequency at each applied field. The frequency range for integration is centered at the resonance frequency and is 250 MHz broad. We hence obtain the frequency-integrated signal I as a function of applied magnetic field. After evaluating I_{max}, i.e. the maximum of I, the field values (critical fields) achieving 30% ($\mu_0 H_{30\%}$), 50% ($\mu_0 H_C$), and 70% ($\mu_0 H_{70\%}$) of I_{max} are extracted.

The entire procedure is conducted by an automated script in MATLAB that iterates the procedure for datasets that are acquired at different P_{irr}. For sufficiently high P_{irr} (e.g. Fig. S4b) the AP branch vanishes for positive fields beyond 0 mT and the P branch is observed starting at 2 mT (Fig. S4b). When this trend is observed, we evaluate $\mu_0 H_{C1}$ to be reduced to 0 mT. Because there is no signal for the AP branch, $\mu_0 H_{30\%}$ and $\mu_0 H_{70\%}$ are evaluated to be reduced to 0 mT as well. In this case the error bar is set to 1 mT which is half of the field step that we applied to sweep the magnetic field from -90 to +90 mT.

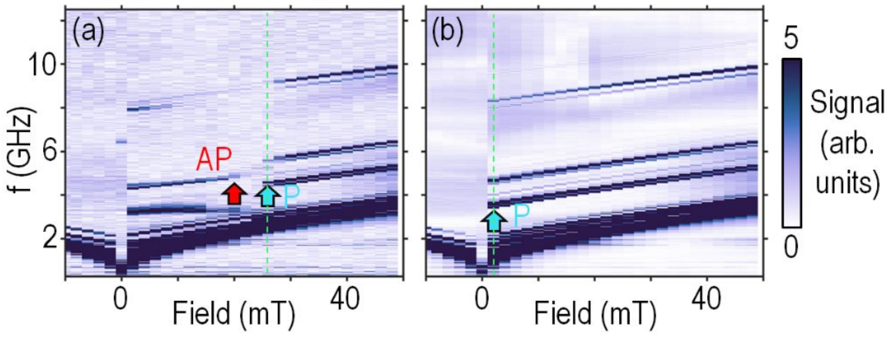

Fig. S4: Mag(S_{21}) transmission spectra for sample B at (a) P_{irr} = -20 and (b) P_{irr} = 0 dBm. (a) The arrows indicate the AP (red) and P (cyan) branches. The dashed green line is drawn as visual guide to indicate the onset of the P branch in each panel.

4. Critical power levels at +20 mT

In Fig. S5 we report an additional experiment characterizing the critical power levels required for switching Py nanostripes beneath CPW1 and CPW2 for both samples A (red circles) with Cu intermediate layer and B (blue diamonds) with SiO_2 intermediate layer. Using the method of switching yield maps, developed in Ref. [1], we characterize the critical power levels (Fig. S5a,b) P_C and (Fig. S5c,d) $P_{C,prec}$ at +20 mT. We analysed the intensity of the vanishing (appearing) branch in the range 4.7-4.9 GHz (4.15-4.4 GHz) to extract P_{C1} (P_{C2}). Following the same approach that we described in the main text (Methods section) we use the reflection spectra acquired at -25 dBm (-10 dBm) to estimate $P_{C1,prec}$ ($P_{C2,prec}$). P_{C1} (Fig. S5 a) and P_{C2} (Fig. S5 b) increase with increasing frequency for both samples. For the main CPW-induced mode, the two samples feature comparable power values. At higher frequencies the critical power levels to initiate switching are lower in sample B than in A. These observations agree with the reported trend at a lower field +14 mT (discussed in the main text). At +20 mT the highest magnon mode in YIG, that we measured leading to switching, is attributed to $k_1 + G + k_{PSSW1}$ (defined in the main text). At +20 mT this mode is observed in both samples. In sample B this mode can lead to switching beneath both CPWs whereas in sample A the $k_1 + G + k_{PSSW1}$ is resolved to initiate switching only beneath CPW1. The GC mode and the $k_1 + G + k_{PSSW1}$ mode require less power in sample B with SiO_2 intermediate layer to initiate switching than in sample A with Cu intermediate layer. $P_{C1,prec}$ (Fig. S5 c) and $P_{C2,prec}$ (Fig. S5 d) at the GC mode frequency have comparable values suggesting weak magnon decay across the two CPWs. These observations match what we reported in the main text for +14 mT (Fig. 4).

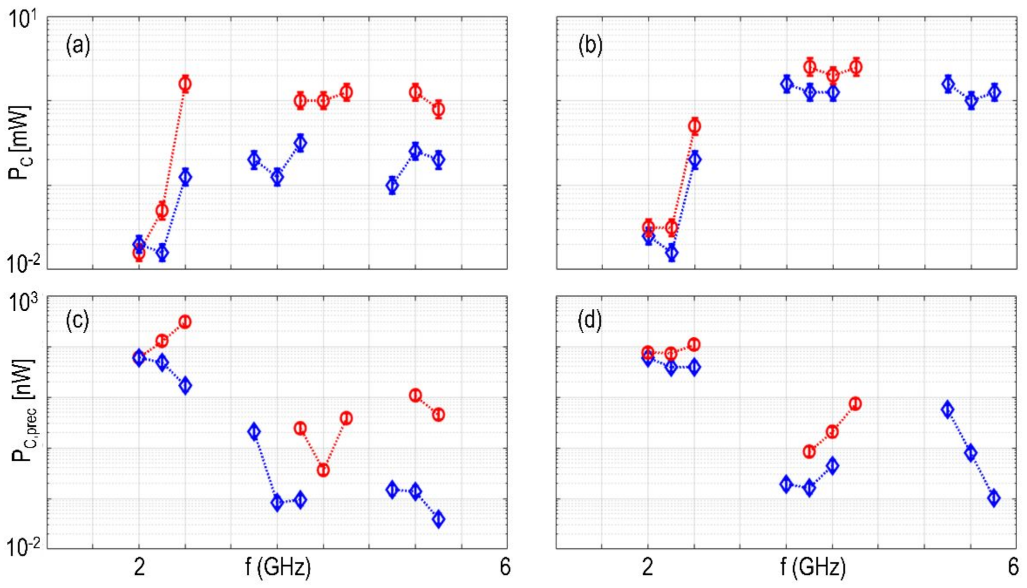

Fig. S5: Critical powers (a) P_{C1}, (b) P_{C2}, (c) $P_{C1,prec}$ and (d) $P_{C2,prec}$ are reported as a function of irradiation frequency for samples A (red) and B (blue) with Cu and SiO_2 intermediate layer, respectively, at +20 mT.

6. Thermal magnon BLS measurements at the emitter CPW (CPW1)

For sample A we conduct BLS measurements at the emitter CPW (CPW1) as illustrated in Fig. S6a. We perform the same measurement protocol that is used to produce the data discussed in the main text. In Fig. S6 we sketch the experiment configuration. Before and after injecting oscillating current into CPW1 the thermal magnon spectrum is measured in positions 1.2 and 1.1. In Fig. S6 we report the results of thermal magnon spectra of Py nanostripes before and after applying an rf signal at 2.3 GHz, 4.3 GHz, 5 GHz and 1.25 GHz. We observed modified spectra when the irradiation frequency matched a magnon resonance. This occurred at 2 mT after irradiating at 1.25 GHz (Fig. S6d,i) and at 24 mT (Fig. S6a-c,f-h). Irradiation at 1.25 GHz for 24 mT did not cause a change in the magnon spectrum of the stripes below CPW1 (Fig. S6j). At this magnetic field, 1.25 GHz was below the magnon band minimum. The modification of the spectra was identified in the vanishing of the peak A and the peak B (see labels) for the spectrum taken before the irradiation and in the appearance of the peak B' in the spectrum taken after irradiation as indicated in Fig. S6. Thermal magnon spectra were modified after application of a radiofrequency signal that either oscillated at a frequency matching a magnon resonance (direct magnon excitation) or a frequency that was twice a magnon frequency thus leading to parametric magnon excitation beyond a certain threshold power. In Fig. S6e we attribute the slight decrease of magnon signal at 2.2 GHz as an effect initiated by the high-power electromagnetic stimulus. The magnetic susceptibility at 1.25 GHz is non-zero hence the magnetization can respond non-resonantly to the rf signal (Fig. S6e). The red curves are all taken after few hours of continuous wave (CW) irradiation at (nominal power) 16 dBm (≈ 39.8 mW). The experiment at 24 mT at 1.25 GHz is used as a control measurement proving that the vanishing of peak A (B) and appearance of peak B' were not caused either by heating or by microwave-assisted switching.

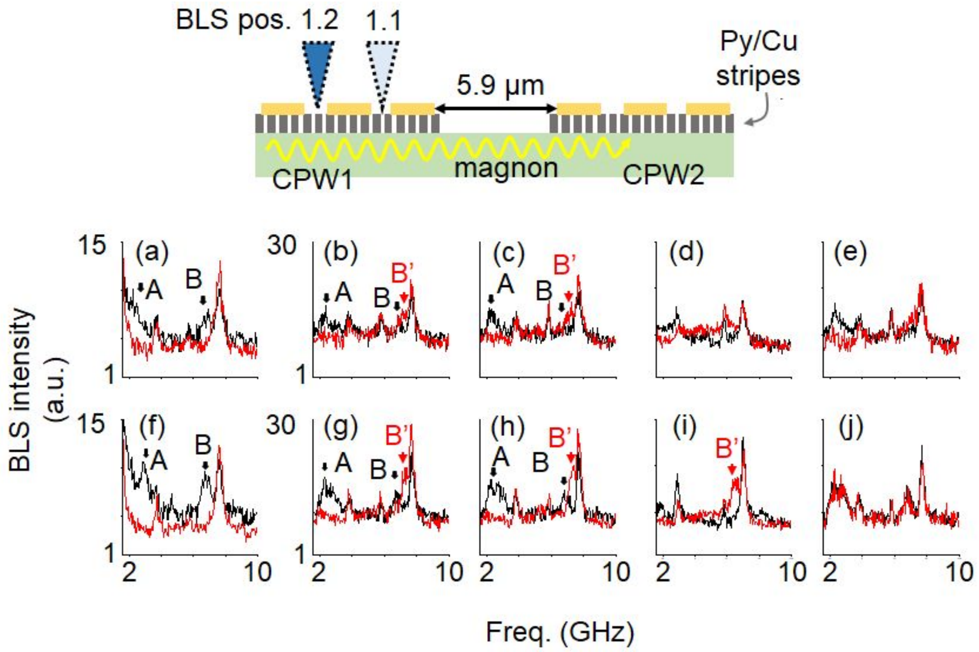

Fig. S6: Sketch of the device and measurement positions and BLS thermal spectra before (after) irradiation are in black (red) curves. (a)-(e) [(f)-(j)] Measurements in Pos 1.1 [Pos 1.2] at 24 mT with frequency 2.3 GHz (a),(f); at 24 mT with frequency 4.3 GHz (b),(g); at 24 mT with frequency 5 GHz (c),(h); at 2 mT with frequency 1.25 GHz (d),(i); at 24 mT with frequency 1.25 GHz (e),(j).

7. BLS measurements during continuous-wave excitation of multiple magnon modes

For sample A we discuss the appearance of parametrically pumped magnons in that we present BLS signals (Fig. S7) acquired in Pos. 2.1 (blue) and Pos. 2.2 (green) *during* continuous wave excitation at f_e = 4.3 GHz (left column) and 5 GHz (right column) for P_{irr} = 0, 10 and 16 dBm at 24 mT. The inelastically scattered photons were accumulated for two hours while the continuous microwave signal was applied. By this approach, both the microwave excited magnons and the magnons incoherently excited by thermal energy were detected. The latter ones gave eventually rise to a magnon peak near 6.9 GHz allowing us to determine the magnetic state of the nanostripes. In each panel the red arrow indicates the peak at the excitation frequency f_e. We observe small peaks at f_e for 0 dBm in Fig. S7(a) and (b). Analyzing the zoomed-in spectra in (g) and (j), respectively, we find that the excitation parameters do not induce a change in the peak near 6.9 GHz (yellow downward triangle). The peak indicates the AP magnetic state of the Py nanostripes with respect to YIG. For P_{irr} = 10 dBm, the signal directly excited at f_e increases in intensity. In Fig. S7(c) [(d)] the increase is up to 25 times [4 times] compared to Fig. S7(a) [(b)]. The black arrow in (c) highlights a parametrically pumped mode at $f = f_e/2$. Note that the blue spectrum in Fig. S7(h) taken at Pos. 2.1 does not contain the peak at the yellow downward triangle indicating that the corresponding Py nanostripes reversed to the P state. The peak is however still present in the green spectrum obtained in Pos 2.2. This observation is explained by the decaying magnon amplitude as the magnons travel further underneath the grating. When exciting at 5 GHz, the power of 10 dBm [Fig. S7(d)] is not enough to change the state of Py nanostripes in Pos. 2.1 and Pos. 2.2 [Fig. S7(k)]. This is different for P_{irr} = 16 dBm [Fig. S7(e) and (f)]. The green-marked peaks are absent in Fig. S7(i) and (l), respectively, for, both, Pos 2.1 and 2.2. At f_e = 5 GHz, 16 dBm is beyond the threshold power for parametric excitation [black arrow in Fig. e)] and $f_e/2$ is close to the k_1

resonance. This mode is known to propagate from CPW1 to CPW2 allowing for Py nanostripe reversal. We have conducted the experiments also for sample B and report similar observations. In sample B we have observed magnon-induced nanomagnet reversal via continuous-wave and pulsed excitation of magnons (Fig. S8).

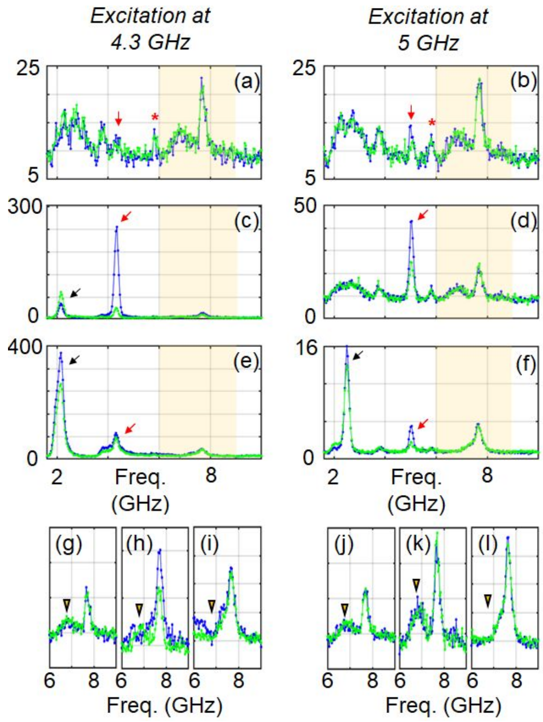

Fig. S7: Spectra obtained at Pos. 2.1 (blue) and Pos. 2.2 (green) with continuous wave RF signal i_{rf} at f_e = 4.3 GHz (left column) and 5 GHz (right). The irradiation power was 0 dBm in (a),(b),(g), and (j); 10 dBm in (c),(d),(h), and (k), and 16 dBm in (e),(f),(i), and (l). In (a) and (b), the red arrows highlight the peaks corresponding to the directly excited modes. The black arrows indicate a parametrically pumped mode, showing up for 10 dBm in (c) and for 16 dBm in (e) and (f). The spectra shown in (g)-(i) for excitation at 4.3 GHz and (j) to (l) at 5 GHz have adjusted y-scales to analyze in detail the peak (highlighted by the triangle) that indicates the magnetic configuration of the Py nanostripes. The marked peak vanishes after the magnon-assisted reversal. The asterisk in (a) and (b) indicates a parasitic peak coming from the laser. The yellow shaded area in (a)-(f) is illustrated with a different y-scale in (g)-(l) to observe peaks with small intensity.

8. Thermal magnon BLS measurements of Py stripes in a device with Py/SiO_2/YIG interface

We have probed magnon-induced nanomagnet reversal in another device with Py/SiO_2/YIG interface, i.e. sample B. Maintaining the same geometry and spacing with each other, we have fabricated Py stripes outside the CPW area (Fig. S8a). The thermal magnon spectra were acquired on different positions on the Py stripes before and after RF irradiation. The RF irradiation was continuous wave (CW) and pulsed. For the CW case we made observations that agreed with our findings in sample A discussed in the main text.

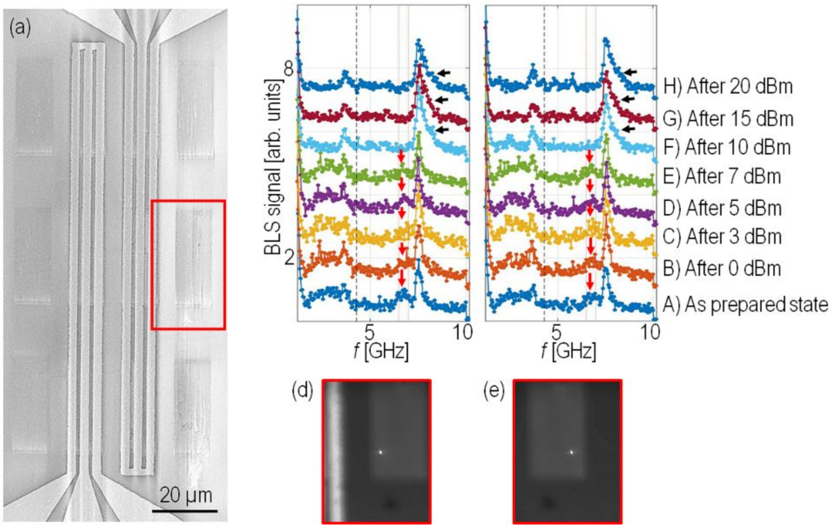

Fig. S8: (a) Scanning electron microscopy image of sample B showing Py stripes outside the CPW area (e.g. red box). (b), (c) BLS thermal magnon spectra at 24 mT acquired on the Py gratings at different distance from the CPW after irradiation at 4.3 GHz for different power levels. The lateral labels (A-H) indicate the history of applied RF signal at 4.3 GHz. The red (black) arrow in each spectrum indicate the Py resonance before (after) the magnon-induced magnetization reversal. (d),(e) Camera images of the BLS measurement position on the Py stripes.

We initialized the system at +90 mT and then increased the field to -24 mT following the same protocol that discussed in the main text. We note that here the sign of the magnetic field was reversed because we targeted to have the magnon with largest amplitude propagating towards the uncovered Py stripes outside the CPW area (red box in Fig. S8a). We recorded thermal magnon spectra in two different positions within the same Py gratings (Fig. S8d,e) of the 'as-prepared' magnetic state that we knew to be AP state from the VNA measurements. With increasing power levels an RF signal oscillating at 4.3 GHz was provided to CPW1. f = 4.3 GHz was the 1st GC mode resonance that corresponded to magnons having a wavelength λ_m = 195 nm. After each power step the RF source was switched off and the thermal magnon spectra were acquired. In Fig. S8b,c we summarize these measurements. An offset is added to each curve for sake of visibilty. The thermal magnon spectra of the AP state (curve A in Fig. S8b,c) are in well agreement with our data obtained on sample A. As the irradiation power is increased above 10 dBm we observed that the spectra are modified. The peak corresponding to the Py nanostripes' resonance in the AP state (red arrows in Fig.s S8b,c) vanishes after irradiation at a power $P \geq 10$ dBm. We observe a new peak (black arrow) after irradiation at $P \geq 10$ dBm. We interpret this as a signature of magnon-induced switching as discussed in the main text.

REFERENCES

[1] Baumgaertl, K. and Grundler, D., 2023. Reversal of nanomagnets by propagating magnons in ferrimagnetic yttrium iron garnet enabling nonvolatile magnon memory. *Nature Communications*, *14*(1), p.1490.
